# Adjuvant hysterectomy following primary chemoradiation for stage IB2 and IIA2 cervical cancer: a retrospective comparison of complications for open versus minimally invasive surgery

**DOI:** 10.1186/s13014-021-01843-0

**Published:** 2021-06-29

**Authors:** Heather Miller, Koji Matsuo, Lynda D. Roman, Annie A. Yessaian, Huyen Q. Pham, Marianne Hom, Antonio Castaneda, Anthony Pham, Omar Ragab, Laila Muderspach, Marcia Ciccone, Laurie L. Brunette

**Affiliations:** 1 Division of Gynecologic Oncology, University of Southern California, 2020 Zonal Ave, IRD 526, Los Angeles, CA 90033 USA; 2 Department of Radiation Oncology, University of Southern California, Los Angeles, CA 90033 USA

**Keywords:** Adjuvant hysterectomy, Cervical cancer, Chemoradiation, Complication, Fistula, Hysterectomy, Vaginal cuff

## Abstract

**Background:**

Adjuvant hysterectomy following chemoradiation for bulky, early stage cervical cancer has been shown to decrease local relapse rate. The objective of this study is to compare complications and recurrences between minimally invasive and open adjuvant hysterectomy for early stage cervical cancer.

**Methods:**

Patients were identified who had undergone adjuvant hysterectomy following chemoradiation for 2009 FIGO stage IB2 and IIA2 cervical cancer from August 2006 to June 2018. Demographic information, treatment course, complications, recurrence data were retrospectively extracted from the medical record. Frequency of complications was compared with Fisher exact test or chi-square test as appropriate and inverse probability of treatment propensity score weighting was used to calculate the disease-free survival.

**Results:**

Fifty-four patients met inclusion criteria with a median follow up time of 60.4 months (interquartile range 28.0–98.1 months). There were 24 (44%) open versus 30 (56%) minimally invasive hysterectomies performed. The overall grade 2 or worse complication rate was 43%. There were 8 (27%) patients with complications in the minimally invasive group compared to 4 (17%) in the open group (OR 1.82 (95% CI 0.5–7.0)). There were 9 vaginal cuff defects, dehiscences and/or fistulas in the minimally invasive group compared to 3 in the open group (OR 3.0 (95% CI 0.8–11.2)). There was no statistically significant difference between disease free survival and overall survival among the two groups, however there was a trend towards decreased disease-free survival in the minimally invasive group.

**Conclusions:**

Among women undergoing adjuvant hysterectomy following chemoradiation for bulky, early stage cervical cancer, there was no difference in complication rates between an open or minimally invasive surgical approach. However, the overall complication rate was high, including a high rate of vaginal cuff defect, dehiscence and/or fistulas. Our findings suggest that an adjuvant hysterectomy should be reserved for patients in which chemoradiation is not anticipated to successfully treat the primary tumor and, if performed, an open approach should be considered.

## Introduction

Concurrent chemoradiation is the standard of care for bulky cervical cancer, which includes 2009 FIGO stage IB2 disease or higher [1]. The benefit of adjuvant hysterectomy following chemoradiation for bulky early stage cervical cancer has been an ongoing source of debate [2–4]. These women have historically high local recurrence rates and it has been suggested that the geometry of these bulky tumors and the associated tumor hypoxia was better addressed by hysterectomy than by additional intracavitary radiation [5]. Studies have shown no difference in overall survival for patients who undergo adjuvant hysterectomy, but patients with large tumors may benefit from the procedure by decreasing local recurrence rates [5–7]. As a result, it had been standard practice at our institution to perform adjuvant hysterectomy for these patients. Historically, this was performed via an open approach, but more recently a minimally invasive surgery with laparoscopy or robotic assistance has been used. No studies have examined the role of minimally invasive surgery compared to open surgery in these patients. Furthermore, recent data has emerged that shows an increase in recurrence rates and overall mortality in minimally invasive surgery versus open surgery for early stage cervical cancer [8, 9]. The objective of this study is to evaluate the complication and recurrence rates for stage IB2 and IIA2 cervical cancer in patients undergoing adjuvant hysterectomy after chemoradiation via minimally invasive surgery compared to open approach.

## Methods

A retrospective cohort study was conducted by retrospective chart review after obtaining institutional review board approval. Strengthening the Reporting of Observational Studies in Epidemiology guidelines were followed.

### Patient selection

All patients at a single, safety net institution with 2009 FIGO stage IB2 and IIA2 cervical cancer from August 2006 to June 2018 were considered for chemoradiation followed by adjuvant hysterectomy. If a patient declined an adjuvant hysterectomy at the time of diagnosis or was deemed to not be a surgical candidate based on comorbidities or performance status, they received definitive chemoradiation. Only those patients who ultimately underwent adjuvant hysterectomy after chemoradiation were included in this study. Only patients with squamous cell carcinoma, adenocarcinoma, or adenosquamous carcinoma were included. Any patient who received additional chemotherapy as part of initial cancer treatment outside of chemosensitization was excluded.

### Treatment details

All patients received a standard external beam radiation dose of 45 Gy and cisplatin for chemosensitization, followed by tandem and ovoid brachytherapy. As all patients were planned to receive an adjuvant hysterectomy following chemoradiation, the final tumor equivalent dose (EQD_2_) of combination external beam radiation and brachytherapy was limited to a total dose to Point A of 75 Gy or less. Most patients also underwent para-aortic lymphadenectomy at the time of hysterectomy for pathologic evaluation of the lymph nodes to determine the need for subsequent extended field radiation. An extrafascial (type 1) hysterectomy was planned for all patients, however, if the surgeon was concerned for gross residual disease in the cervix based on preoperative exam, a modified radical or radical (type II or III) hysterectomy was performed in order to achieve a complete resection of the primary tumor with an adequate margin. Medical records were retrospectively reviewed to extract data on patient demographics, cancer treatment, surgical details, complications and recurrence.

### Endpoint definitions

The primary outcome was overall complication rate, calculated as total number of complications per total number of patients. Some patients had multiple complications. Secondary outcomes included disease free survival and overall survival. Only grade 2 or worse complications at the time of surgery and up to 90 days postoperatively were recorded, based on the classification of surgical complications [10]. Only complications related to the surgical procedure were recorded, therefore excluding toxicity from radiation therapy or chemosensitization. Date and status at last follow up was recorded until April 2020. Patients who were alive at last follow up were censored on that date.

### Statistical analysis

Frequency of perioperative complications was compared between the minimally invasive surgery and open group with Fisher exact test or chi-square test as appropriate. Effect size was expressed with odds ratio (OR) and 95% confidence interval (CI).

The second step of the analysis was to assess the outcome measures (OS and DFS) related to type of surgery performed. The propensity score inverse probability of treatment weighting (PS-IPTW) was used to balance the measured covariates between the two groups [11]. This analytic approach was used due to small sample size with limited survival events in which a multivariate analysis would result in over adjustment. PS-IPTW approach is superior to PS-matching as the matching process will further reduce the sample size that likely increases the risk of type II error. PS-IPTW modeling is also superior to conventional adjustment model in such limited sample size as multiple adjusting factors may result in overadjustment. [11].

The PS was computed by fitting a multivariable binary logistic regression model for the type of surgical approach. Given the small sample size, stringent covariates selection was deployed by choosing a priori historical factors reporting survival effects in cervical cancer (histology type, cancer stage, and residual disease on hysterectomy specimen) [12–14].

The PS-IPTW approach assigned women in the MIS group a weight of 1/PS and women in the laparotomy group a weight of 1/(1-PS), respectively. Size effect was assessed between the two groups in the weighted model, and a standardized difference of > 0.20 was considered a presence of size effect with clinical imbalance [15]. In a PS-IPTW model, Kaplan-Meier method was used to plot survival curves, and a Cox proportional hazard regression model was fit to estimate survival effect of MIS use, expressed with hazard ratio (HR) and 95% CI.

In a sensitivity analysis, the effect of surgical approach on survival was examined in the unweighted model. All analyses were based on two-tailed hypothesis, and a *P* < 0.05 was considered statistically significant. Statistical Package for Social Sciences (version 25.0, Armonk, NY, USA) and R version 3.5.3 (R Foundation for Statistical Computing, Vienna, Austria) were used for analyses.

## Results

### Patient characteristics

Fifty-four patients met inclusion criteria for the study and their demographic information is shown in Table 1. There were 24 (44%) open versus 30 (56%) minimally invasive hysterectomies performed. The majority, 49 (91%), were extrafascial (type 1) and 5 (9%) were radical (type III) or modified radical (type II) hysterectomies. Radical or modified radical hysterectomy was performed if there was concern for gross residual disease on exam on the day of surgery. The majority of patients also had pelvic and/or paraaortic lymphadenectomy performed at the time of hysterectomy (22 (92%) patients in the open surgery group versus 28 (93%) patients in the minimally invasive surgery group). The study population had a median age of 44 years (interquartile range 40–52) and 74% were of Hispanic ethnicity. There were no statistically significant differences between groups with regard to age, race, ethnicity, body mass index or cigarette use. The tumor histology was predominantly squamous in both groups. Twenty-one (88%) of patients in the open surgery group and 29 (97%) in the minimally invasive group were diagnosed with FIGO 1 stage IB2 disease. Median tumor size at diagnosis was 6.4 cm (interquartile range 5.0–7.0 cm) in the open group compared to 6.2 cm (interquartile range 5.0–7.0 cm) in the minimally invasive surgery group. Median doses of chemosensitization with cisplatin was 5 (range 3–6). Following external beam radiation, 25 (46%) patients received low dose rate brachytherapy, and 29 (55%) patients received high dose rate brachytherapy. The average radiation dose to Point A in patients undergoing adjuvant hysterectomy was 74.4 Gy.

**Table 1:** Demographics

	Surgical approach		
	Open	Minimally invasive	P-value
Number	n = 24	n = 30	
Age (y)	44 (IQR 39–49)	44 (IQR 40–56)	0.79
*Race*			0.19
White	18 (75.0%)	24 (80.0%)	
Black	4 (16.7%)	1 (3.3%)	
Asian	2 (8.3%)	5 (16.7%)	
*Ethnicity*			0.76
Hispanic	17 (70.8%)	23 (76.7%)	
Non-Hispanic	7 (29.2%)	7 (23.3%)	
*Year*			< 0.01
2006–2011	22 (91.7%)	2 (6.7%)	
2012–2018	2 (8.3%)	28 (93.3%)	
BMI (kg/m2)	27.6 (IQR 25.2–31.4)	29.3 (IQR 25.0-35.4)	0.44
*Cigarette use*			0.77
No	21 (87.5%)	27 (90.0%)	
Yes	3 (12.5%)	3 (10.0%)	
*Histology*			0.19
Squamous	16 (66.7%)	17 (56.7%)	
Adenocarcinoma	4 (16.7%)	11 (36.7%)	
Adenosquamous	4 (16.7%)	2 (6.7%)	
*Clinical Stage*			0.2
IB2	21 (87.5%)	29 (96.7%)	
IIA2	3 (12.5%)	1 (3.3%)	
*Grade*			0.13
1	1 (4.2%)	8 (26.7%)	
2	5 (20.8%)	7 (23.3%)	
3	13 (54.2%)	10 (33.3%)	
Not defined	5 (20.8%)	5 (16.7%)	
Tumor size on exam (cm)	6.4 (IQR 5.0–7.0)	6.2 (IQR 5.0–7.0)	0.87
*Type of hysterectomy*			0.46
Type I	21 (87.5%)	28 (93.3%)	
Type II or III	3 (12.5%)	2 (6.7%)	

Median,* IQR* interquartile range

Twenty-two (92%) patients treated from 2006 to 2011 underwent open surgery, whereas after this, 28 (93%) patients from 2012 to 2018 underwent a minimally invasive surgery (p < 0.01). Adjuvant hysterectomy was performed at a median time of 8.0 weeks following completion of chemoradiation (range 3.9–15.6 weeks). There was no difference in the median time to adjuvant hysterectomy between patients who experienced complications and those who did not: median time 8.2 weeks (interquartile range 7.1–9.7 weeks) and 8.3 weeks (interquartile range 7.1–9.9 weeks), respectively. Following adjuvant hysterectomy, 33 (61%) patients had residual disease in their hysterectomy specimen with median residual disease size of 14.5mm (interquartile range 6.8-27.8mm). In all of the minimally invasive hysterectomies, the vaginal cuff was sutured from within the peritoneal cavity using an intracorporeal or extracorporeal laparoscopic or robotic technique.

### Complications

The median follow-up time was 60.4 months (interquartile range 28.0-98.1 months) for the entire cohort. In the MIS group the median follow-up time was 42.0 months (interquartile range 30.0–78.0 months) and 85.2 months (interquartile range 37.2–126.0 months) in the open group. Twelve of the 54 patients (22%) experienced at least one grade 2 or worse complication attributable to the hysterectomy: 8 (27%) in the minimally invasive group and 4 (17%) in the open group (OR 1.8, 95% CI 0.5–7.0, p = 0.35). The details of each of these complications are listed in Table 2. Among these 12 patients, there were 23 total grade 2 or worse complications for an overall complication rate of 43%, summarized in Table 3. Three (25%) had received low dose rate and 9 (75%) high dose rate brachytherapy. Nine of these patients (75%) had residual disease in their cervix on final hysterectomy pathology, compared to 24 of the 42 patients (57%) who did not experience a grade 2 or higher surgical complication (OR 2.3, 95% CI 0.5–9.5, p = 0.27). Ten patients experienced at least one complication involving the vaginal cuff, for a total of 12 complications involving the vaginal cuff such as a vaginal cuff defect, dehiscence and/or fistula between the vagina and urinary or gastrointestinal tract. Nine of these vaginal cuff complications were noted in the minimally invasive group compared to 3 in the open group (OR 3.0, 95% CI 0.8–11.2, p = 0.12).

**Table 2:** Details of patients experiencing grade 2 or worse surgical complications

Patient #	Type	Surgical approach	Complication	Treatment
1	Extrafascial	Open	Vaginal cuff necrosis	Conservative management
2	Modified radical	Open	Vaginal cuff dehiscence	Conservative management
			Acute blood loss anemia	Blood transfusion
			Vesicovaginal fistula	Percutaneous nephrostomy tubes
3	Extrafascial	Open	Urinary tract infection	Antibiotics
4	Extrafascial	Open	Ureteral injury	Intraoperative repair
			Acute blood loss anemia	Blood transfusion
			Wound seroma	Conservative management
5	Extrafascial	Laparoscopic	Vaginal cuff dehiscence	Vaginal cuff repair
			Acute blood loss anemia	Blood transfusion
			Port site hematoma	Conservative management
6	Extrafascial	Laparoscopic	Vaginal cuff necrosis	Conservative management
7	Extrafascial	Laparoscopic	Vaginal cuff dehiscence	Vaginal cuff repair
8	Extrafascial	Laparoscopic	Vaginal cuff dehiscence	Conservative management
9	Modified radical	Robotic	Ureterovaginal fistula	Ureteroneocystotomy
			Sepsis	Antibiotics
10	Extrafascial	Laparoscopic	Vaginal cuff dehiscence	Vaginal cuff repair
			Acute blood loss anemia	Blood transfusion
			Sepsis	Antibiotics
11	Extrafascial	Robotic	Vaginal cuff dehiscence	Conservative management
			Urinary tract infection	Antibiotics
12	Extrafascial	Laparoscopic	Vaginal cuff dehiscence	Conservative management
			Rectovaginal fistula	Diverting loop colostomy

**Table 3:** Complication rates overall and by surgical approach

	Overall	Surgical approach	
		Open	Minimally invasive
Number of patients	54	24	30
Number of patients experiencing any complications	12 (22%)	4 (17%)	8 (27%)
Total number of complications (rate (%))*	23 (43%)	8 (33%)	15 (50%)
Blood loss requiring transfusion	4 (7%)	2 (8%)	2 (7%)
Port site hematoma	1 (2%)	0 (0%)	1 (3%)
Wound seroma	1 (2%)	1 (4%)	0 (0%)
Urinary tract infection	2 (4%)	1 (4%)	1 (3%)
Sepsis	2 (4%)	0 (0%)	2 (7%)
Ureteral injury	1 (2%)	1 (4%)	0 (0%)
Vaginal cuff complication or fistula	12 (22%)	3 (13%)	9 (30%)

*Complication rate calculated as total number of complications per total number of patients. Some patients experienced multiple complications

Of the 3 patients who developed fistulas following adjuvant hysterectomy, 2 were noted to have a vaginal cuff dehiscence prior to the fistula diagnosis. One patient developed a ureterovaginal fistula that was managed with primary surgical repair with ureteroneocystotomy. One patient developed a vesicovaginal fistula following conservative management of a vaginal cuff dehiscence that was treated with bilateral percutaneous nephrostomy tubes initially for symptomatic relief following which she was lost to follow up. The third patient developed a rectovaginal fistula after conservative management of a vaginal cuff dehiscence and subsequently underwent a diverting loop colostomy.

There was higher estimated blood loss, 250 mL versus 88 mL (p < 0.001), in the open group compared to minimally invasive surgery, as well as increased median length of stay [5 vs. 3 days (p < 0.01)] (Table 4). There were 6 (20%) patients with multiple complications in the minimally invasive group compared to 2 (8%) in the open group.

**Table 4:** Complications by surgical approach

	Open (n = 24)	Minimally invasive (n = 30)	P-value	Odds ratio (95% confidence interval)
Patients experiencing any complication	4 (17%)	8 (27%)	0.52	1.82 (0.47–6.97)
*Complications per patient*				
No complications	20 (83%)	22 (73%)	0.49	
Single complication	2 (8%)	2 (7%)		
Multiple complications	2 (8%)	6 (20%)		
Estimated blood loss (ml)	250 (IQR 156–350)	88 (50–105)	< 0.001	
Length of hospital stay (days)	5 (IQR 4–6)	3 (IQR 2–3)	< 0.001	

### Recurrence and survival outcomes

During the follow up period there were 9 recurrences and 7 deaths, 6 from cervical cancer disease progression and 1 which was non-cancer related. There were 3 (13%) recurrences in the open group and 6 (20%) in the minimally invasive group (OR 1.75, 95% CI 0.39–7.88, p = 0.47). Of the 9 recurrences, 2 (22%) had local recurrences at the vaginal cuff, 6 (67%) had distant recurrences, including nodal recurrences and 1 (11%) had both local and distant recurrence. The PS-IPTW post weighted statistics for each group based on stage, histology and residual disease are shown in Table 5. Of the 33 patients with residual disease in their hysterectomy specimen, 8 (24.2%) had a recurrence compared to 1 recurrence among the 21 patients (4.8%) who did not have residual disease in their hysterectomy specimen (OR 6.4, 95% CI 0.74–55.5, p = 0.09). All 3 of the patients who underwent open surgery and experienced a recurrence had residual disease in their hysterectomy specimen compared to 5 of the 6 who underwent MIS and had a recurrence.

**Table 5:** Postweighted balance statistics

	Open (%)	MIS (%)	P-value	SD (pre-IPTW)	SD (post-IPTW)
*Clinical Stage*			0.93	0.35	0.03
IB2	7.3	6.6			
IIA2	92.7	93.4			
*Histology*			0.99	0.29	0.01
Squamous	60.0	59.9			
Adenocarcinoma	28.9	28.8			
Adenosquamous	11.1	11.3			
*Residual cervical disease*			0.98	0.28	0.06
None	40.3	39.7			
Microscopic	37.8	36.1			
Macroscopic	22.0	24.3			

*MIS *minimally invasive surgery, *SD *standardized difference, *IPTW *inverse probability of treatment weighting

**Fig. 1:**
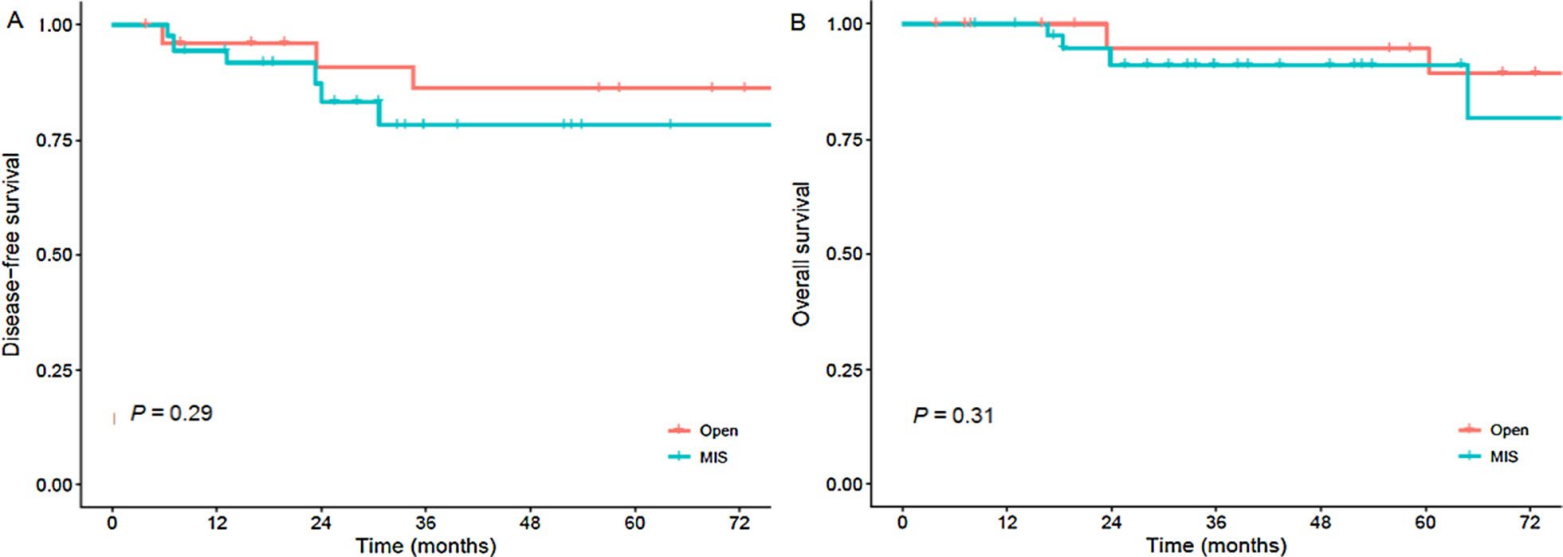
**A** Disease free survival, **B** overall survival. *MIS *minimally invasive surgery

Disease free survival using PS-IPTW showed no differences between groups and was 86.3% in the open group compared to 78.3% in the minimally invasive group (HR 1.71 (0.64–4.61) p = 0.29). Similarly, there were no differences in 5-year overall survival between groups using PS-IPTW and was 94.6% in the open group compared to 91.0% in the minimally invasive group (HR 1.89 (0.55–6.49) p = 0.31), Fig. 1.

## Discussion

In 2003, a phase III randomized trial enrolled 256 patients with stage IB2 cervical cancer and compared those who underwent adjuvant hysterectomy versus no hysterectomy after primary chemoradiation [5]. Although not specifically noted in the manuscript, it can be inferred that these were performed via the open approach as the study was performed before the minimally invasive era. They reported combined complication rates from radiation therapy and surgery, but noted that the addition of the hysterectomy did not increase the rate of complications, with an overall greater than grade 3 complication rate of 10%^5^. Our study found an overall grade 2 or greater complication rate of 43% for adjuvant hysterectomies in this setting, but no statistically significant difference between patients undergoing minimally invasive compared to open surgery.

There were more vaginal cuff complications in patients undergoing minimally invasive compared to open hysterectomy; and multiple patients required surgical repair of the dehiscence or management of a resulting fistula. Albeit not statistically significant, likely due to the small sample size, this trend is clinically significant given the severity of this complication. Prior studies have examined risk factors for vaginal cuff dehiscence, with mode of hysterectomy playing an important role. Retrospective studies have shown a rate of vaginal cuff dehiscence of 1.1–4.1% for laparoscopic hysterectomy and 3% for robotic hysterectomy. This is compared to a rate of 0.29% for vaginal hysterectomy and 0.12% for abdominal hysterectomy [16, 17]. Type of hysterectomy also affects vaginal cuff dehiscence rates with radical hysterectomy via minimally invasive surgery showing a 9-fold increase in vaginal cuff complications compared to extrafascial hysterectomy [17]. Transvaginal closure of the cuff has also been shown to have a 3- to 9-fold decrease in dehiscence rate compared to laparoscopic closure [18]. The reason for increased vaginal cuff dehiscence in laparoscopic hysterectomies is not fully known but theories have been postulated. During minimally invasive hysterectomy the colpotomy is made with electrocautery, which may lead to more tissue necrosis and poor wound healing, something which is particularly concerning in an irradiated population. Also, the method of closing the vaginal cuff via laparoscopic or vaginal suturing may be inferior to the open technique and lead to worse outcomes. In our study, all patients had a malignancy and received radiation therapy, which are known risk factors for vaginal cuff complications and poor wound healing [19]. It is likely that the combination of these risk factors and the increased risk of cuff complications with minimally invasive hysterectomies results in a particularly high rate of vaginal cuff necrosis and dehiscence.

While not statistically significant, there was a trend towards shorter disease-free survival in the group undergoing minimally invasive adjuvant hysterectomy. This parallels recent data from the laparoscopic approach to cervical cancer trial showing a decrease in progression-free survival and increased mortality in patients undergoing primary hysterectomy by minimally invasive compared to open approach [8]. Other studies have shown similar results, including a large surveillance, epidemiology and end results (SEER) database study that showed a decrease in overall survival in patients undergoing minimally invasive surgery compared to open radical hysterectomy [9]. The mechanism by which minimally invasive surgery may lead to increased mortality and recurrence rates in cervical cancer patients remains unknown. It has been postulated that use of the uterine manipulator facilitates intraperitoneal tumor spread. It is also possible there is tumor spill into the peritoneal cavity when making the colpotomy that plays a role in this process. In our cohort, 61% of patients had residual disease at time of adjuvant hysterectomy. This is attributed to the lower doses of brachytherapy these patients received in anticipation of undergoing a hysterectomy and is in line with prior studies [5]. While not statistically significant, there was a trend towards higher recurrence rates among patients with residual disease in their hysterectomy specimen in both the open surgery and MIS groups, but the low number of events limits further analysis of this by surgical approach.

Radiation and surgical techniques have evolved substantially since the clinical trial published in 2003 that reported a lower incidence of local relapse in patients receiving adjuvant hysterectomy compared to those who did not after chemoradiation [5]. More recently, a phase III trial was conducted in France that compared the role of adjuvant hysterectomy in stage IB2 and II cervical cancer. This study was closed early due to poor accrual, but ultimately analyzed 30 patients who received adjuvant hysterectomy and 31 patients who received chemoradiation only. The findings were not statistically significant but showed improved disease-free survival and overall survival in patients who did not undergo adjuvant hysterectomy [20]. It is possible, especially in the era of modern radiation oncology, that there is limited clinical benefit and increased morbidity with routine performance of an adjuvant hysterectomy. Thus, adjuvant hysterectomy should be reserved for situations when intracavitary brachytherapy is not feasible or the response from chemoradiation is deemed unlikely to result in sterilization of the primary tumor.

A weakness of our study is the small sample size. However, data was collected over a 12-year period and due to the specific study population there are few studies that include more patients. A potential confounding factor in our study was the year in which surgery was performed. The decision on route of surgery was at the discretion of the surgeon, but from 2006 to 2011 nearly all patients received an open hysterectomy, whereas after 2011 the majority of patients underwent minimally invasive surgery. Given this, the follow up time for the open cases is longer than minimally invasive surgery. This bias has the potential to mask possible future recurrences in the minimally invasive group, which could lead to greater differences in recurrence rates and possibly overall survival. Additionally, other factors may have changed over this time period that affected the outcome, such as changes in surgeon experience and the learning curve associated with adopting new minimally invasive surgical techniques.

There was also a change in type of brachytherapy given to patients during the study window. From 2006 to 2011, the majority of patients received low dose rate brachytherapy after completion of external beam radiation. In contrast, from 2012 to 2018, the majority of patients received high dose rate brachytherapy. Low dose rate brachytherapy delivers a radiation dose of 0.4–2 Gy/h whereas high dose rate brachytherapy delivers the dose much faster at > 12 Gy/h. The switch from low dose rate to high dose rate brachytherapy was made at a very similar time as the switch from open to minimally invasive hysterectomy at our institution. Therefore, most of the patients who underwent minimally invasive adjuvant hysterectomy also received high dose rate brachytherapy. Of the 12 patients with complications following adjuvant hysterectomy, 3 (25%) had received low dose rate and 9 (75%) high dose rate brachytherapy. It is possible the change in radiation also contributed to the increased rate of complications seen, however it is not possible to make definite conclusions regarding this given our small sample size and confounding factors.

While older studies do show a decreased local recurrence rate by adding adjuvant hysterectomy to radiation for these patients [5], the improvement in radiation techniques and addition of cisplatin chemosensitization since that time may make these findings no longer relevant as is suggested by our study and others [20–22].

## Conclusions

Among women undergoing adjuvant hysterectomy following chemoradiation for bulky, early stage cervical cancer, there was no difference in complication rates between an open or minimally invasive surgical approach, however, the overall complication rate was high, including a high rate of vaginal cuff defect, dehiscence and/or fistulas. Our findings suggest that an adjuvant hysterectomy should be reserved for patients in which chemoradiation is not anticipated to successfully treat the primary tumor and, if performed, an open approach should be considered given the trend towards fewer complications and improved survival noted in this group compared to those undergoing a minimally invasive surgery.

## Data Availability

The data that support the findings of this study are available from the corresponding author, [LLB], upon reasonable request.

## References

[1] Vale C, Tierney JF, Stewart LA (2008). Reducing uncertainties about the effects of chemoradiotherapy for cervical cancer: a systematic review and meta-analysis of individual patient data from 18 randomized trials. J Clin Oncol.

[2] Rutledge FN, Wharton JT, Fletcher GH. Clinical studies with adjunctive surgery and irradiation therapy in the treatment of carcinoma of the cervix. *Cancer*. 1976;38(1):596–602. 10.1002/1097-0142(197607)38:1<596::AID-CNCR2820380184>3.0.CO;2-C 10.1002/1097-0142(197607)38:1<596::aid-cncr2820380184>3.0.co;2-c 1277111

[3] O’Quinn AG, Fletcher GH, Wharton JT (1980). Guidelines for conservative hysterectomy after irradiation. Gynecol Oncol.

[4] Mendenhall WM, McCarty PJ, Morgan LS, Chafe WE, Million RR (1991). Stage IB or IIA-B carcinoma of the intact uterine cervix ≥ 6 cm in diameter: is adjuvant extrafascial hysterectomy beneficial?. Int J Radiat Oncol Biol Phys.

[5] Keys HM, Bundy BN, Stehman FB, et al. Radiation therapy with and without extrafascial hysterectomy for bulky stage IB cervical carcinoma: a randomized trial of the Gynecologic Oncology Group. Gynecol Oncol. 2003;89:343–53. 10.1016/S0090-8258(03)00173-2. 10.1016/s0090-8258(03)00173-2 12798694

[6] Decker MA, Burke JJ, Gallup DG (2004). Completion hysterectomy after radiation therapy for bulky cervical cancer stages IB, IIA, and IIB: complications and survival rates. Am J Obstet Gynecol.

[7] Rema P, Suchetha S, Kumar A, Ahmed I (2015). The Role of Adjuvant Hysterectomy After Radiotherapy in Cervical Cancer. Indian J Surg.

[8] Ramirez PT, Frumovitz M, Pareja R (2018). Minimally Invasive versus Abdominal Radical Hysterectomy for Cervical Cancer. N Engl J Med.

[9] Barber EL, Rauh-Hain JA, Rice LW (2019). Survival After Minimally Invasive Radical Hysterectomy for Early-stage Cervical Cancer. Obstet Gynecol Surv.

[10] Dindo D, Demartines N, Clavien PA (2004). Classification of surgical complications: a new proposal with evaluation in a cohort of 6336 patients and results of a survey. Ann Surg.

[11] Austin PC, Stuart EA (2015). Moving towards best practice when using inverse probability of treatment weighting (IPTW) using the propensity score to estimate causal treatment effects in observational studies. Stat Med.

[12] Mabuchi S, Okazawa M, Matsuo KM (2012). Impact of histological subtype on survival of patients with surgically-treated stage IA2-IIB cervical cancer: adenocarcinoma versus squamous cell carcinoma. Gynecol Oncol.

[13] Matsuo KM, Shimada M, Saito T, et al. Risk stratification models for para-aortic lymph node metastasis and recurrence in stage IB-IIB cervical cancer. J Gynecol Oncol. 2018;29(1):e11. 10.3802/jgo.2018.29.e11. 10.3802/jgo.2018.29.e11 PMC5709521 29185269

[14] Kunos C, Ali S, Abdul-Karim FW, et al. Posttherapy residual disease associates with long-term survival after chemoradiation for bulky stage 1B cervical carcinoma: a Gynecologic Oncology Group study. Am J Obstet Gynecol. 2010;203(4):351.e1–8. 10.1016/j.ajog.2010.05.005. Epub 2010 Jun 11. 10.1016/j.ajog.2010.05.005 PMC2947558 20541170

[15] Muller K, Cohen J. Statistical power analysis for the behavioral sciences. Technometrics. 1989. 10.2307/1270020.

[16] Hur HC, Guido RS, Mansuria SM, Hacker MR, Sanfilippo JS, Lee TT (2007). Incidence and patient characteristics of vaginal cuff dehiscence after different modes of hysterectomies. J Minim Invasive Gynecol.

[17] Nick AM, Lange J, Frumovitz M (2011). Rate of vaginal cuff separation following laparoscopic or robotic hysterectomy. Gynecol Oncol.

[18] Uccella S, Ceccaroni M, Cromi A (2012). Vaginal Cuff Dehiscence in a Series of 12,398 Hysterectomies. Obstet Gynecol.

[19] Wiebe E, Covens A, Thomas G (2012). Vaginal vault dehiscence and increased use of vaginal vault brachytherapy: what are the implications?. Int J Gynecol Cancer.

[20] Morice P, Rey A, Rouanet P, et al. Results of a Phase III FNLCCC trial comparing hysterectomy versus no hysterectomy after chemoradiation therapy in IB2/II stage cervical cancer. Int J Gynecol cancer. 2011;21(12 SUPPL. 3 CC-Gynaecological, Neuro-oncology and Orphan Cancer):S203. 10.1097/IGC.0b013e318235bd21.

[21] Tanderup K, Lindegaard JC, Kirisits C (2016). Image Guided Adaptive Brachytherapy in cervix cancer: a new paradigm changing clinical practice and outcome. Radiother Oncol.

[22] Keys HM, Bundy BN, Stehman FB (1999). Cisplatin, radiation, and adjuvant hysterectomy compared with radiation and adjuvant hysterectomy for bulky stage IB cervical carcinoma. N Engl J Med.

