# Correction to: Adjuvant hysterectomy following primary chemoradiation for stage IB2 and IIA2 cervical cancer: a retrospective comparison of complications for open versus minimally invasive surgery

**DOI:** 10.1186/s13014-021-01881-8

**Published:** 2021-08-25

**Authors:** Heather Miller, Koji Matsuo, Lynda D. Roman, Annie A. Yessaian, Huyen Q. Pham, Marianne Hom, Antonio Castaneda, Anthony Pham, Omar Ragab, Laila Muderspach, Marcia Ciccone, Laurie L. Brunette

**Affiliations:** 1 Division of Gynecologic Oncology, University of Southern California, 2020 Zonal Ave, IRD 526, Los Angeles, CA 90033 USA; 2 Department of Radiation Oncology, University of Southern California, Los Angeles, CA 90033 USA

## Correction to: Radiat Oncol (2021) 16:123 https://doi.org/10.1186/s13014-021-01843-0

Following publication of the original article [1], the authors identified an error in the author name of Koji Matsuo.

- The incorrect author name is:  Koji M. Matsuo
- The correct author name is: Koji Matsuo

The author group has been updated above and the original article [1] has been corrected.
